# Life in a World without Microbes

**DOI:** 10.1371/journal.pbio.1002020

**Published:** 2014-12-16

**Authors:** Jack A. Gilbert, Josh D. Neufeld

**Affiliations:** 1 Institute for Genomic and Systems Biology, Argonne National Laboratory, Argonne, Illinois, United States of America; 2 Department of Ecology and Evolution, University of Chicago, Chicago, Illinois, United States of America; 3 Marine Biological Laboratory, Woods Hole, Massachusetts, United States of America; 4 College of Environmental and Resource Sciences, Zhejiang University, Hangzhou, China; 5 Department of Biology, University of Waterloo, Waterloo, Ontario, Canada

## Abstract

Life in a world without microbes Can a macrorganism survive without commensal microbes in a world of microbes? What would happen if all microbes on earth suddenly disappeared?

*“Life would not long remain possible in the absence of microbes.”—Louis Pasteur*

Or would it?

How many times have we started proposals, manuscripts, or presentations with compelling statements about the critical roles that microorganisms play in sustaining life? How often has the possibility of a world without microbes been explored in our introductory microbiology classes? Within the human microbiome research community, entire fields explore the interdependence of humans and their microbial counterparts.

But what would happen in a world without microbes?

In order to promote discussion about the value of microbial services supporting life on this planet, we explore the opportunities and challenges of a microbe-free existence. Our discussion begins by considering life without the human gut microbiome, follows with a hypothetical scenario of a world without Bacteria and Archaea, and concludes with the implications of a world without all microbes, including microbial eukaryotes and viruses. We do not include the organelles, such as mitochondria and chloroplasts, as microbes in our discussion, simply because most eukaryotic life would cease instantly in their absence.

We argue that despite myriad fundamental roles that microorganisms contribute to human and environmental function, it would be false to claim that macroscopic life cannot exist without microbes. However, although life would persist in the absence of microbes, both the quantity and quality of life would be reduced drastically.

## Gnotobiotic Life

The concept of animals existing in complete isolation from microorganisms originated with Louis Pasteur [1], who also predicted that an animal's existence would be impossible without microbial life. Ten years later, George Nuttal and Hans Thuerfelder disproved Pasteur's prediction by removing microorganisms from a guinea pig [2]. Much later, James Reyniers and colleagues reared rats and chickens in gnotobiotic conditions (*gnos*, known; *bios*, life; i.e., “germfree”), enabling the development of germfree animal populations for research [3],[4]. Reynier's bioengineering-driven efforts to generate “pure units” of biology for experimental study resulted in technology that enabled gnotobiotic life. Thus began not only a field of scientific endeavor that would alter the face of medical and biological study but also a cultural phenomenon centered on an obsession with eliminating microorganisms from the human experience, with extremes leading to “germophobia.”

The gnotobiotic condition has often been purported to enable an animal to enjoy improved physiological health, even leading to an increased life span. Misinterpreted reports from early 20th century research propagated the misconception that animals, including humans, might thrive without microbes, producing healthier children and adults [5]. However, such generalizations are oversimplified. Although the absence of microorganisms, pathogens included, does tend to increase lifespan [6], germfree animal physiology and immunology are altered, with poorly characterized consequences. Gnotobiotic animals have reduced motility in the bowel that results in a greatly enlarged cecum, which can lead to lethal complications [7]. In addition, these animals possess smaller lymph nodes and a poorly developed immune system, including reductions in serum immunoglobulin and leukocytes. Germfree animals also exhibit reduced organ sizes, including for the heart, lungs, and liver. Certain other aspects of gnotobiotic development have not been rigorously examined. For example, gnotobiotic conditions may have unforeseen consequences on mental health due to the myriad interactions between the gut microbiome and neurophysiological health and development [8],[9].

Although animal life can survive without direct physical contact with Bacteria and Archaea, are microorganisms necessary for generating the nutritional requirements, dietary supplements, and foodstuffs required for metabolism? Indeed, early experiments in gnotobiotic systems resulted in nutrition-related deaths because microorganisms associated with these animals produced growth factors essential to the host [5]. Today, such nutritional issues have largely been solved. Animals can spend their entire lives absent of microbial flora because all required dietary components can be synthesized chemically, without the need for a biological precursor.

Despite the possibility of meeting nutritional requirements for a human germfree existence, perhaps the most substantial barrier for our species embracing a gnotobiotic lifestyle is this: who would want to live inside a bubble? Without the commensal microbes that colonize our bodies and train our immune systems, sudden exposure to pathogenic microorganisms would likely result in a disease burden that would shorten our lifespans dramatically. A bubble would be essential for maintaining gnotobiotic life in our current world, as it was for David Vetter [5]. The physiological and psychological consequences of rearing a human being to adulthood under gnotobiotic conditions are entirely unknown.

## Bacteria and Archaea

What if we could live a germfree life outside the bubble? What if all prokaryotic microorganisms on Earth disappeared suddenly? If someone were to wave an antimicrobial wand and eliminate all bacterial and archaeal life on the planet, what would happen? The usual rhetoric is that life as we know it would end, human societies would collapse, and eukaryotic life would cease to exist. Is all of this true?

These same questions were asked by Moselio Schaechter as part of the “Talmudic Questions” series of the *Small Things Considered* blog [10]. David Lipson's subsequent response focused correctly on the immediate problem of nitrogen. Plants require fixed nitrogen, and bacteria play an essential biological role in the fixation process. Lipson suggests that, without help from humans, most global photosynthesis would cease within a year. Humans could potentially increase synthetic fertilizer production via the Haber-Bosch process and initiate a massive global fertilization scheme, alleviating some of the enormous losses of life. Such human intervention would be facilitated, to some extent, by the absence of bacterial denitrification and anaerobic ammonia oxidation, which would otherwise deplete fixed nitrogen. Ultimately, nitrogen would begin to accumulate in the global oceans. One possibility is that life would distribute along the coasts, where N-rich fish could be harvested and fixed nitrogen scavenged from seawater when atmospheric nitrogen depletion, due to the Haber-Bosch process, exhausted atmospheric reserves.

Unfortunately, the inevitable increase in atmospheric CO_2_ concentration due to animal respiration and human fossil fuel use would lead to rapid global warming through the greenhouse effect. Lipson points out that the process would require hundreds of years to eliminate life on the planet—ample time to find a carbon capture solution? In this way, some degree of agricultural food production and marine photosynthesis could continue indefinitely, supporting a subset of humans. Nonetheless, the world's oceans and soils would likely begin a process of stagnation due to the myriad absent contributions to global biogeochemistry.

What about humans and our ability to breathe? How much of global atmospheric oxygen is accounted for by bacterial activity? Oxygenic photosynthetic progenitors transformed the world's atmosphere from anoxic to oxic during the Great Oxygenation Event, beginning approximately 3,000,000,000 years ago [11]. *Prochlorococcus* and *Synechoccocus* are now two of the world's most abundant cellular life forms, filling the ocean to varying degrees from pole to pole, generating oxygen as a byproduct of sunlight-driven photosynthesis. If these great oxygen sources vanished from the world's oceans, lakes, surface soils, and plant surfaces, then what would happen? Perhaps surprisingly, it is unlikely that anything problematic for aerobic life would happen for at least a few hundred thousand years. Assuming humans could distribute nitrogen globally, algae and plants could be expected to continue generating a proportion of available atmospheric oxygen, potentially as high as 50% [12]. Existing pools of atmospheric oxygen might satisfy the demand for aerobic metabolism among surviving organisms, possibly for decades or centuries. If this were the case, then asphyxiation of aerobic life would not be likely in the near term.

What about all the accumulating waste? For example, in a world free of Bacteria and Archaea, the most immediately impacted entities would be bacteriophage (i.e., viruses that prey on host bacteria) and archaeal viruses, which would likely disassociate without their coevolved hosts. With an estimate of ∼1×10^30^ phage in the world [13], one wonders what the release of so much carbon, phosphorous, and nitrogen contained in their DNA, RNA, and capsid proteins would do to global ecosystems and biogeochemistry. Perhaps more importantly, prokaryotic biomass represents roughly one-half of all global biomass. If the antimicrobial wand did not result in the actual disappearance of these cells, then waste bacterial and archaeal cells would further contribute to biomass decomposition problems. Would the higher microbial life forms (e.g., aerobic and anaerobic fungi and protists) be able to decompose and assimilate it sufficiently? Whereas insects, microscopic animals, protists, slime molds, and fungi do much of the initial biomass decomposition for material recycling, Bacteria and Archaea contribute unique and essential roles for completing the task, especially under anoxic conditions (e.g., anaerobic respiration, interspecies hydrogen transfer, and methanogenesis).

Biomass would likely begin to accumulate, particularly at the molecular level, creating vast reservoirs of biogeochemical waste that no biological entity could transform, at least initially. This would lead to the eventual disruption of the biogeochemical recycling upon which all life ultimately depends and a gradual return of these persistent compounds to geological material. For example, phosphorous would begin to disappear, given that it is a nonrenewable element. The ocean would become virtually nonproductive, possibly within decades, without the regeneration of phosphorous in the water column. Phosphorous sequestration to sediments would impact marine primary production, which would be difficult to offset sustainably by anthropogenic inputs, especially given an eventual depletion of phosphorous mines.

Another consideration is that most living organisms must complement their diet with bacterial and archaeal cofactors and enzymatic activity. For example, without Bacteria and Archaea, ruminants (e.g., cows, sheep, and goats) would be almost completely unable to derive benefit from a cellulose-heavy diet in the absence of nutritional intervention by human chemists. Although humans depend on microbial vitamins and amino acids obtained through diet or our gut microorganisms, we might successfully synthesize nutritional compounds through chemical ingenuity or by recombinant biotechnology with yeast as a surrogate host. Other organisms would have less potential for human intervention. For example, termites and their anaerobic protists depend on bacterial and archaeal symbionts for their metabolism. Moreover, more than half of all phytoplankton require vitamin B_12_ from bacterial partners [14]. As such, many eukaryotes, including termite and phytoplankton species, would likely expire by nutrient and cofactor starvation in a world deprived of Bacteria and Archaea.

In summary, most global biogeochemical cycling would grind to a halt in a world without Bacteria and Archaea; humans would need to fix and distribute nitrogen for maintaining crop production. Fungal decomposition would become the critical link between organismal death and decay and the return of decomposed nutrients to the bottom of the eukaryotic food chain. Most species on Earth would become extinct, and population sizes would be reduced greatly for the species that endured.

How long would it take for humans to notice what had happened? Surprisingly, humans would fail to see many signs of this global change for a few days or weeks. We could still digest our food, as do gnotobiotic animals, assimilating most of what we consumed. We would still battle viral, fungal, and parasitic infections. Even though our dairy industries, cattle farmers, biotechnology companies, food producers, hospitals, and wastewater treatment systems would begin making headlines within a day or two, it would take us nearly a week to realize what had happened. We predict complete societal collapse only within a year or so, linked to catastrophic failure of the food supply chain. Annihilation of most humans and nonmicroscopic life on the planet would follow a prolonged period of starvation, disease, unrest, civil war, anarchy, and global biogeochemical asphyxiation.

## Microbes

If the antimicrobial wand were waved, this time removing all microbes (i.e., viruses, Bacteria, Archaea, fungi, and protists—algae and others) from the planet, what would happen next?

One of the very first observations in a world without all microbes would be a shocking absence of all forms of microbial disease, including Ebola, malaria, the common cold, ulcers, *Clostridium difficile*, and athlete's foot, to name a few. This complete freedom from microbial illness would be welcomed, initially, by jubilant media headlines announcing a global microbiological “miracle.” How long would it take for the celebrations to cease?

If all microbes were to disappear, the future of life on the planet would parallel a world without Bacteria and Archaea (i.e., calamitous; see above), except that the myriad environmental impacts would be more acute. Even more so than in a Bacteria- and Archaea-free world, most biogeochemical cycling would cease; human and animal waste would accumulate rapidly. There would be very little decomposition apart from disassociation and inherent catabolic enzymatic activity. The essential role that microbes play in biomass recycling would not be served even by fungi or protists, resulting in a rapid exhaustion of available macronutrients and micronutrients in terrestrial and aquatic environments. Living food sources would be increasingly difficult to find. As described earlier, most ruminant livestock would starve without microbial symbionts, and plants would rapidly deplete nitrogen, cease photosynthesis, and then die. Intensive human intervention required to produce and distribute sufficient vitamins, plant fertilizers, and food sources would likely overwhelm ingenuity in the face of mounting environmental, ecological, and humanitarian disaster. As with a Bacteria- and Archaea-free world, small pockets of humans and other animals (e.g., insects) would survive for a time, decades or centuries even, but long-term survival of most eukaryotes would be doubtful.

## Conclusion

Microbes sustain life on this planet because of their myriad associations and biogeochemical processes. Nonetheless, their roles are not necessarily irreproducible. When you next hear someone claim that we cannot live without microorganisms, it would be appropriate to ask them to qualify the statement. Would we still be able to eat and digest food? Yes. Would life be extinguished in the absence of Bacteria and Archaea or in a world without any microbes? Not immediately, not all life, and potentially not for a long time.

In short, we argue that humans could get by without microbes just fine, for a few days.* Although the quality of life on this planet would become incomprehensibly bad, life as an entity would endure.

* If we do include mitochondria and chloroplasts as Bacteria, as we should, then the impact would be immediate—most eukaryotes would be dead in a minute.
